# Pooling resources for universal health coverage

**DOI:** 10.2471/BLT.20.020220

**Published:** 2020-02-01

## Abstract

Progress towards universal health coverage in the Caribbean will require greater collaboration between the island states and territories. Gary Humphreys reports.

Hurricane Dorian hit the Bahamas on 1 September 2019. Two days of intense winds, rain and surf devastated the archipelago’s water and communications systems and destroyed many health facilities. Five days later, when the Bahamian authorities were still trying to assess the damage, they received US$5.5 million, the first of two payments from the Caribbean Catastrophe Risk Insurance Facility (CCRIF). 

Created in 2007 and funded by the Caribbean Community or CARICOM, a group of 15 of the 30 states and territories that comprise the Caribbean region, the CCRIF is a testament to the islands’ capacity to collaborate.

“The CCRIF is an excellent example of what the Caribbean states can achieve when they come together in the face of a shared challenge,” says Dr James Hospedales, adjunct clinical professor at Tulane University School of Public Health & Tropical Medicine in the United States of America.

Until August last year, Hospedales was the executive director of another joint effort in the region to pool resources for the common good: the Caribbean Public Health Agency.

The agency combines activities that were previously undertaken by five separate regional health institutions, providing public health services and support to its members that some might otherwise struggle to afford.

Since 2013, when the agency was established, it has ramped up disease surveillance and vector control activities in the region, reinforced the Caribbean’s public health laboratory network, launched a pan-Caribbean Regulatory System for pharmaceuticals, established a register of clinical trials involving human participants and, in June 2018, it launched a cancer registry hub for CARICOM members.

While the Caribbean Public Health Agency has achieved much in the six years of its existence, it has no mandate to work on regional health system strengthening, although this is now a Caribbean Cooperation in Health priority that is explicitly linked to achieving universal health coverage in the region.

To date, health system strengthening remains the preserve of the individual governments, which are moving towards universal health coverage at different rates.

According to *Primary health care on the road to universal health coverage: 2019*, WHO’s most recent universal health coverage monitoring report, coverage of essential health services in the Caribbean ranges from a low of 47% (in Haiti) to 77% (in Barbados), with most of the 15 CARICOM states and territories at or around 70% coverage.

Out-of-pocket payments made by the patient at the point of receiving health care remain relatively high in all states and territories, representing around a third of total health expenditure in the region globally.

“Despite all the talk about universal health coverage, it has not been a political priority.”

Rudolph Cummings.

“Even where public health service coverage is relatively good, many patients prefer to seek care in the private sector and may incur significant costs because service delivery is not always perceived to be of high quality and there are sometimes long waiting times,” says Dr Rufus Ewing, an expert on health systems and services at the Pan-American Health Organization (PAHO) office in Bridgetown, Barbados.

“This happens, for example, in Barbados, where you have a range of health services that are officially free at the point of care, but where out-of-pocket payment remains around 40%,” Ewing adds.

Expanding service coverage and improving health service delivery depends in large part on investing more in public health, which in turn depends on political commitment at the highest level.

Caribbean states have made numerous commitments to developing universal health coverage in recent years, most recently at the United Nations General Assembly high-level meeting on the subject in New York in September 2019, but not everyone is convinced.

“Despite all the talk about universal health coverage, it has not been a political priority,” says Dr Rudolph Cummings, the manager of the Health Sector Development Programme at CARICOM headquarters in Georgetown, Guyana.

Increasing resources for health is a challenge for Caribbean states and territories not least because of their low levels of tax collection and relatively large informal economies, in which workers are harder to tax and harder to draw into health insurance schemes.

Despite the challenges, many governments are moving forward with health system financing initiatives. For example, prior to Hurricane Dorian, the Bahamian government had begun financing the primary health care phase of a national insurance programme, and – once normal business resumes – will probably be funding the second phase, at least in part, with mandatory health insurance for employees, supplemented by a tax on sugary drinks.

However, resource pooling in a small country like the Bahamas is challenging, because, put simply, small pools are easily drained, and one or two years of heavy expenditure can break the bank.

According to Cummings there has been some discussion of territories making use of reinsurers, who would themselves create and insure a bigger risk pool, or even of coming together in joint pooling arrangements, but so far no concrete initiatives have emerged.

For Ewing, moving towards universal health coverage in the Caribbean depends not just on increased investment and sustainable financing, but the reorientation of health systems away from curative services towards preventive and primary health care.

Trevor Hassell, president of the Healthy Caribbean Coalition, a civil society organization dedicated to tackling noncommunicable diseases agrees. “We continue to build too many hospitals,” he says, “and we need to rethink service delivery to cover the full continuum of care, including health promotion, disease prevention, and put primary health care at the centre of our health systems.”

“Noncommunicable diseases will impose an increasing burden on the region’s health systems and budgets,” Hassell says.

Estimates vary, but it is widely accepted that, based on current trends, around 25% (11.7 million/47 million) of the total population will be older than 60 by 2050, up from around 14% (6 million /43 million) today, while the incidence of diabetes, ischaemic heart disease and asthma are increasing, along with associated risk factors, such as obesity and hypertension.

While preventive and primary care is the priority, Caribbean health systems also need to develop specialized curative services if the sustainable development goal universal health coverage target is to be achieved. Many Caribbean states and territories lack their own facilities and pay to send patients abroad for care.

“People in the Caribbean have always had to travel to receive health care.”

James Hospedales

“People in the Caribbean have always had to travel to receive health care,” says Hospedales. “There are about 129 inhabited islands and it’s obvious you’re not going to have health infrastructure in all of them. So to get specialized care you get on a plane to Nassau or Miami.”

In many cases governments pay for citizens’ travel and treatment, incurring costs which Ewing considers to be one of the two greatest threats to the financial sustainability of the region’s health systems (the other being the rising costs of treatment for noncommunicable diseases).

In the English-speaking Caribbean, Barbados, Jamaica and Trinidad have tended to perform the role of specialist care provider, and more recently Grand Cayman has developed Health City Cayman Islands, a tertiary care facility providing a range of services from adult and paediatric cardiology and surgery, to neurosurgery.

So some capacity is being developed, but accessing it is difficult, especially for the poor and there is no regional entity to match needs for service to payment.

“How to cover the needs of citizens who island hop to seek care has long been a topic of discussion in the Caribbean,” says Jessie Schutt-Aine, PAHO’s sub-regional programme coordinator in the Caribbean.

An important aim of previous development strategy was encouraging the movement of skilled labour between the islands, and for that to become reality it was understood that individuals who decided to move needed to be able to access education and health services – and that these services were in fact a right.

Work on a protocol of contingent rights started in 2008 and was finally ready for signature in 2018. By February 2019 all CARICOM member states had signed. But so far only Barbados has drawn up national policy that effectively implements the protocol.

For Hassell, the lack of progress towards the integration and collaboration promised by the Caribbean Single Market and Economy is emblematic of inertia in other areas. “Institutional and policy mechanisms are in place that are the basis for moving forward, but there is an implementation deficit,” he says.

PAHO’s Ewing takes heart from recent developments in the Organisation of Eastern Caribbean States, where collaboration on medicines procurement is already a reality. He also considered the development of the Caribbean Regulatory System to be an important step towards universal health coverage in the area of medicines. 

**Figure:**
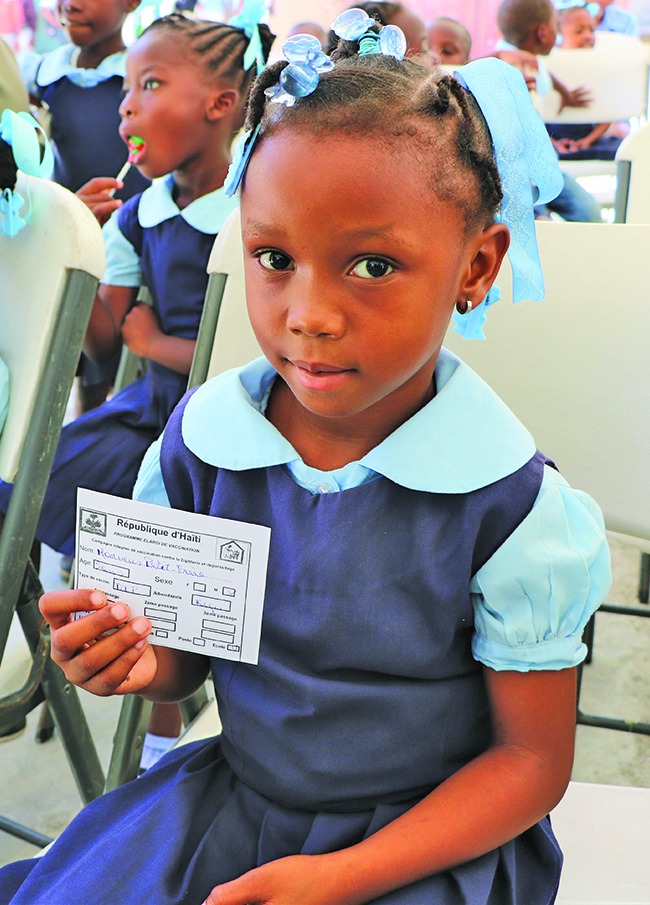
School children are vaccinated and receive their vaccination certificates in Haiti

PAHO

**Figure:**
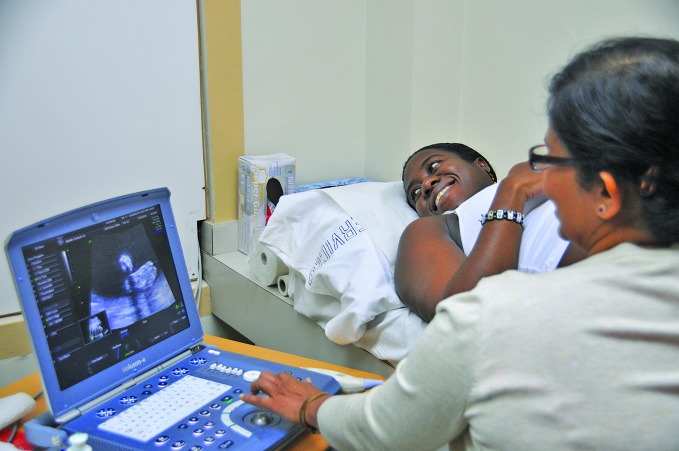
Pre-natal care in Trinidad and Tobago

PAHO

